# Direct Measurements of Electroviscous Phenomena in Nafion Membranes

**DOI:** 10.3390/membranes10110304

**Published:** 2020-10-25

**Authors:** David Nicolas Østedgaard-Munck, Jacopo Catalano, Anders Bentien

**Affiliations:** Department of Engineering, Århus University, Åbogade 40, 8200 Aarhus N, Denmark; dni@liqtech.com

**Keywords:** electroviscous effect, ion exchange membranes, apparent solution viscosity, electrokinetic energy conversion

## Abstract

Investigation of electroviscous effects is of interest to technologies that exploit transport of ions through ion exchange membranes, charged capillaries, and porous media. When ions move through such media due to a hydrostatic pressure difference, they interact with the fixed charges, leading to an increased hydraulic resistance. Experimentally this is observed as an apparent increase in the viscosity of the solution. Electroviscous effects are present in all electrochemical membrane-based processes ranging from nanofiltration to fuel-cells and redox flow batteries. Direct measurements of electroviscous effects varying the applied ionic current through Nafion membranes have, to the best of the authors’ knowledge, not yet been reported in literature. In the current study, electroviscous phenomena in different Nafion ion exchange membranes are measured directly with a method where the volume permeation is measured under constant trans-membrane pressure difference while varying the ion current density in the membrane. The direct measurement of the electroviscous effect is compared to the one calculated from the phenomenological transport equations and measured transport coefficients. Within the experimental uncertainty, there is a good agreement between the two values for all membranes tested. We report here an electroviscous effect for all Nafion membranes tested to be $\kappa_{H}^{🟉}\kappa_{H}^{-1}=1.15_{-0.052}^{+0.035}$.

## 1. Introduction

Electrokinetic phenomena in membranes, charged capillaries, and porous media arise due to electrostatic interactions between mobile ions, polar water molecules, and immobile charges within the ion channels or pores [1,2,3,4]. One consequence of these interactions can be observed in a setup where a pressure gradient forces an electrolyte solution through a membrane with electrodes on each side. If the electrodes are short circuited a net ion charge can be transported through the membrane and the volumetric flow will be larger than the one observed when the electrodes are open circuited. Historically, this flow increase has been defined as the electroviscous effect and described as an apparent decrease of the viscosity of the solution [5,6,7,8]. Electroviscous effect measurements have previously been used to determine surface potentials [6,7,8,9,10,11].

**Figure 1:**
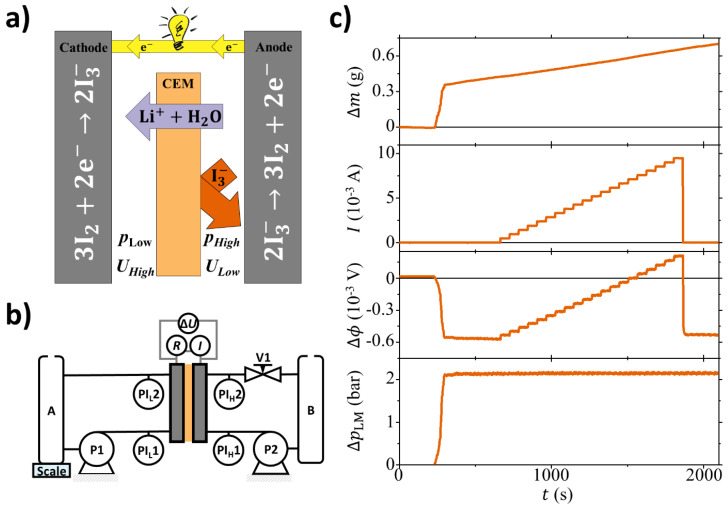
(**a**) Illustrative figure showing the electrokinetic energy conversion (EKEC) principle considering an aqueous LiI/I_2_ electrolyte solutions having equal concentration on both half-cells. In this configuration the high pressure ($p_{\mathrm{High}}$) and the low pressure ($p_{\mathrm{Low}}$) sides are separated by a cation exchange membrane (CEM). (**b**) Schematic of the apparatus used for the experiments. Two peristaltic pumps (P1 and P2) connect the reservoirs (A and B) to each half-cell of the electrochemical device. The pressure is measured at the inlet (PI${}_{H1}$ and PI${}_{L1}$) and outlet (PI${}_{H2}$ and PI${}_{L2}$) of each half cell. Finally, the electrochemical cell is connected to a variable load/source (*R*) to control the current (*I*) while measuring the electrical potential difference. (**c**) Typical set of raw data, from the top to the bottom: (i) Mass change (Δm) in the left-hand side (low pressure side) of the system. (ii) Electrical current (*I*) through the external load. (iii) Electrical potential difference (Δϕ) between the flow cell electrodes. (iv) Logarithmic mean pressure difference: $\Delta p_{\mathrm{LM}}=\left(\Delta p_{1}-\Delta p_{2}\right){\left(\ln\Delta p_{1}-\ln\Delta p_{2}\right)}^{-1}$ where $\Delta p_{1}$ and $\Delta p_{2}$ are the pressure differences at each end of the flow cell (e.g., $\Delta p_{1}=p_{H,1}-p_{L,1}$). Data noise is lower than 0.4 mg, 0.01 mA, 0.003 mV, 0.06 bar, respectively.

Besides electroviscosity, many other electrokinetic phenomena can be observed. Some well-knowm phenomena include, for example, electrophoresis, electro-osmosis, and streaming potential [12]. In the context of energy harvesting, the streaming potential and streaming current can be exploited by utilizing electrokinetic energy conversion (EKEC) where electrodes collect the generate electricity. Figure 1a illustrates the EKEC concept, where a cation exchange membrane (CEM) has aqueous LiI/I_2_ electrolyte solutions of equal concentration on both sides. A pressure gradient ($\Delta p=p_{\mathrm{high}}-p_{\mathrm{low}}$) drives Li^+^ ions and H_2_O molecules through the membrane while most I_3_^−^ are being rejected due to the membrane permselectivity [13]. In order to maintain charge neutrality, the cation transport is balanced by the electrode reaction: 2I_3_^−^ ⇌ 3I_2_ + 2e^−^, thus generating an electrical current (*I*) passing through an external electrical load (represented as a light bulb in Figure 1a). EKEC is reversible, meaning instead of a pressure gradient, an electrical current can be applied to the system. In this case, migrating ions drag coupled polar H_2_O molecules through the membrane, whereby the system functions as an electrokinetic pump. The potential and current generated by (or applied to) the flow cell can be directly related to the electroviscous effect as an increase in flow with increasing current [6,7,8,14,15].

The theoretical maximum EKEC efficiency, $\eta_{\max}$ is defined by the electrokinetic figure-of-merit (β) originally derived from the phenomenological transport equations by Morrison and Osterle [1] and later explored in a different, yet equivalent, form by some of the authors of the present work [14,16]. $\eta_{\max}$ is given by $\eta_{\max}=\left(\sqrt{\beta +1}-1\right){\left(\sqrt{\beta +1}+1\right)}^{-1}$, where β can be expressed in several equivalent ways being $\beta =\nu^{2}\sigma \kappa_{H}^{-1}$ the most relevant for the current work [1,3,14]. Here $\kappa_{H}$ and ν are the hydraulic permeability and streaming potential coefficient, both measured at zero current density (I=0 and Δp≠0) and σ the ion conductivity measured at zero pressure difference (I≠0 and Δp=0).

In previous studies we have determined ν, σ, and $\kappa_{H}$ experimentally in ion exchange membranes to evaluate β, hence indirectly $\eta_{\max}$ [16,17,18,19,20]. This was later followed by a direct measurement of $\eta_{\max}$ [21]. Nonetheless, as pointed out earlier [16,17] it is possible to evaluate the maximum theoretical EKEC efficiency from $\beta =\kappa_{H}^{🟉}\kappa_{H}^{-1}-1$ where $\kappa_{H}^{🟉}$ is the intrinsic hydraulic permeability without electroviscous effects: i.e., measured at nil electrical potential difference across the electrodes. The ratio $\kappa_{H}^{🟉}\kappa_{H}^{-1}$ is defined as the electroviscous effect [14]. With reference to Figure 1a, $\kappa_{H}$ is measured open-circuited, which is the standard condition for the measurement of hydraulic permeability, whereby when the net electrical current (*I*) is zero. In this case, electrolytes that do not permeate couple to the solvent, hence hinder transport and lower the overall permeation. This is due to the open-circuited conditions and permselectivity of the membrane. On the other hand, $\kappa_{H}^{🟉}$ is measured short-circuited, whereby the potential difference (Δϕ) is zero. In this case the cations freely permeate through the membrane along with the solvent, thus resulting in a streaming current and a higher permeability. Qualitatively the relation $\beta =\kappa_{H}^{🟉}\kappa_{H}^{-1}-1$ can thereby be explained as a measurement of the coupling between the solvent and electrolyte in a specific membrane. It is important to note that parasitic resistances in the cell decrease the measured $\kappa_{H}^{🟉}$ and the intrinsic permeability in this case is related to the membrane, cell, and electrode charge transfer resistances [21].

The current paper reports a detailed study of the electroviscous effect in Nafion membranes by measurement of the volumetric flow ($q_{V}$) as a function of the streaming current that is varied by an external electrical load. In particular, the range between I=0 and Δϕ=0, which defines $\kappa_{H}$ and $\kappa_{H}^{🟉}$, respectively, is extensively investigated. These experiments are performed on four different membranes: Nafion212 (N212), Nafion117 (N117), cast pure Nafion (NC), and cast Nafion modified (N-SPS30) by using sulfonated polystyrene (SPS) as a sacrificial porogen and a solution containing LiI/I_2_ (0.3 and 1 M) redox pair. With the selected LiI/I_2_ redox pair, carbon paper or felt, normally adopted in flow-battery systems, can be used as electrodes. This choice greatly simplifies the experimental setup with respect to, for example,: Ag/AgCl meshes. Measurement of $q_{V}$ as a function of the streaming current in low permeability ion exchange membranes has not yet been reported in the literature. This is presumably due to experimental challenges associated with both the measurement of $q_{V}$ and the construction of optimized electrochemical cells that minimize the extrinsic electrical resistance. Lowering the ohmic/ionic resistances not related to the membrane, is a prerequisite for observing the electroviscous effects in this type of membranes. On the other hand these phenomena will be relevant in any optimized electro-membrane system exploiting the ion movement to generate or store electricity.

## 2. Materials and Methods

### 2.1. Preparation of Solutions and Membranes

The LiI/I_2_ electrolyte solutions were made by using Lithium Iodide, LiI (Sigma-Aldrich: Lithium Iodide Hydrate, 223816) in Milli-Q Water and saturated with Iodine, I_2_ (Sigma-Aldrich: Iodine, 229695). The aqueous LiI/I_2_ solutions were prepared immediately before use, and the reservoirs were protected from direct light with aluminium foils. Nafion117 (dry thickness: 178 μm), Nafion212 (dry thickness: 50 μm), and Nafion solution (ion exchange capacity (iec) = 0.95 to 1.01 meq g${}^{-1}$) were acquired from Fuel Cell Store (https://www.fuelcellstore.com/). The cast membranes were synthetized by following the approach described by Kristensen et al. [22]. Briefly: 2 g of Nafion were dissolved in 30 mL Dimethylacetamide (DMAc, anhydrous 98.9%, Sigma Aldrich) and sulfonated polystyrene (SPS, 30% *w*/*v* aqueous solution, MW 75,000, iec${}_{\mathrm{SPS}}=5.4$ meq g${}^{-1}$, Alfa Aesar) was added. SPS was pretreated by drying and re-dissolved in DMAc before use. After stirring, the membranes were cast in petri dishes to cure for ∼40 h at room temperature. Afterward, the membranes were dried under vacuum at 25 °C and then rinsed in milli-Q water. The extruded and cast membranes were chemically pretreated prior to use as in Reference [22]: the membranes were pretreated by boiling in H_2_O_2_ (3 wt%), then H_2_SO_4_ (1 M), and finally in Milli-Q water. The membranes were stored in 1 M LiCl until use (at least 24 h) to obtain the Li^+^ form. In the present paper Nafion membranes without any additives (NC) and Nafion membranes with 30 wt% (with respect to Nafion) Sulfonated Polystyrene (N-SPS30) were cast.

### 2.2. Electrochemical Flow Cell

The electrochemical flow cell consists of two symmetrical half cells sandwiching the membrane having a geometrical active area 25 cm${}^{2}$, a stainless-steel endplate, a sheet of insulating Teflon, a golden-plated copper current collector, isomolded graphite with machined interdigitated flow pattern, an O-ring, and a 0.1 mm piece of carbon paper electrode (Fuel Cell Store: Toray Carbon Paper, pretreated in air at 500 °C for 7 h). Each half-cell has four ports; one connected to each corner of the square flow pattern. Two diagonally opposite ports conduct the solution flow in and out while the remaining two ports are connected to pressure indicators (PI, Druck DE, range 1–10 bar). This ensures a pressure reading of inlet and outlet of both cell halves thus enabling the calculation of the logarithmic mean pressure difference: $\Delta p_{\mathrm{LM}}=\left(\Delta p_{1}-\Delta p_{2}\right){\left(\ln\Delta p_{1}-\ln\Delta p_{2}\right)}^{-1}$ where $\Delta p_{1}$ and $\Delta p_{2}$ are the pressure differences at each end of the flow cell (e.g., $\Delta p_{1}=p_{H,1}-p_{L,1}$). A similar flow cell has been previously described in more detail in Reference [13].

Figure 1b illustrates the experimental setup. Each cell half is connected to a reservoir (A and B, 250 mL each ) through a peristaltic pump (P1 and P2, Cole-Parmer: HV-07554-85, HV77250-62). The system was set up to run the flow cell halves in counter flow. The right-hand side of the system is equipped with a needle valve (V1), such that the electrolyte solution circulated as follows: B → P2 → PI${}_{H1}$→ cell → PI${}_{H2}$→ V1 →B. This enables pressure control of the right-hand side while monitoring the pressure drop across the cell flow pattern. The solution path of the left-hand side is similar, with the difference being no installed valve and reservoir A being placed on a microscale (Sartorius: Quintix 224-1S, accuracy 0.1 mg). The scale monitors the mass change (Δm) in the left-hand side of the system, i.e., its derivative versus time is related to the volume permeation through the membrane from the right-hand side of the system. The electrodes are connected through an electrical load/source unit (Agilent U2722A, full scale ± 120 mA) controlling the current (*I*) and thus contrary to Reference [21] it works in the four quadrants varying the electrical load/source. The potential across the membrane (Δϕ) is monitored by an NI analog input module (National Instruments: NI 9219). All data are logged with LabView (National Instruments) at 0.5 Hz.

A combined volume of 500 mL LiI/I_2_ solution was used for each experiment. The relatively large volumes ensure that the LiI/I_2_ concentration on each side does not change significantly during an experiment. Initially the solution was flushed through both cell halves for approximately 30 min, ensuring the exact same concentration on both sides of the membrane. Then the solution was split into the two reservoirs A and B. Peristaltic pumps circulated solutions at a flow rate ∼0.2 mL s${}^{-1}$ until stable baselines for all parameters (namely Δm, *I*, Δϕ, and $\Delta p_{\mathrm{LM}}$) were recorded. Then valve V1 was engaged to pressurize the right-hand side of the flow cell, resulting in signal changes of all parameters but *I*. Initially the system was left to stabilize for a few minutes before *I* was ramped up from 0 to 10 mA, in steps of 0.5 mA. At each current measurements proceeded for 60 s. All the experiments were carried out at room temperature ( 21 ± 1 °C).

## 3. Results and Discussion

The single most important contribution to the experimental noises and uncertainties is related to fluctuations of the mass-measurement due to the peristaltic pumps. Much lower noise can be achieved by reducing the pump circulation flow rate ($q_{\mathrm{circ}}$). However, in this case a non-intrinsic concentration polarization may affect the experiments [21]. To minimize concentration polarization phenomena electrochemical impedance spectroscopy (EIS) was employed at different $q_{\mathrm{circ}}$ and the most optimal flow rate was chosen based on a trade-off between minimized impedance in the diffusion limited regime and minimized noise in the mass measurement. We have earlier reported the effect of $q_{\mathrm{circ}}$ between 0.2 and 3.2 mL s${}^{-1}$ on the impedance in the diffusion limited regime for Nafion117 [21]. For the present work $q_{\mathrm{circ}}$ 0.2 mL s${}^{-1}$ was chosen even though it meant a decrease in apparent ion conductivity ($\sigma^{🟉}$). In particular, for Nafion212, by increasing the flow rate from $q_{\mathrm{circ}}=0.2$ to 3.2 mL s${}^{-1}$, the apparent ion conductivity increased from $\sigma^{🟉}$ = 0.3 to $\sigma^{🟉}=0.4$ S m${}^{-1}$ (seen from EIS). This in turn affects the total cell resistance and, indirectly, the magnitude of the observed electroviscous effect. Nonetheless, for Nafion212 membranes, we observed a smaller decrease in $\sigma^{🟉}$ compared to Nafion117 while the diffusion limited regime is still not prominent; furthermore, a reduction of the circulation flow rate of almost 20 times markedly increased the resolution of the volumetric flow measurements. Based on this observation, we are confident that the measured permeation rates are intrinsic to the membranes and concentration polarization only has a small influence.

Figure 1c shows a set of raw data from a single experiment done with Nafion212 and 1 M LiI/I_2_ solution. The system is stabilizing until t∼250 s where a pressure difference of ∼2 bar is applied across the flow cell. This is followed by a sudden increase of Δm of around 400 mg due to the pressure-induced membrane deformation. Afterward, a steady, linear increase related to pressure-driven permeation is seen. For Δϕ a similar sudden change is observed and it settles within minutes at a constant value around −0.6 mV. This is a direct measure of the streaming potential coefficient: $\nu ={\left[\Delta \phi \Delta p^{-1}\right]}_{I=0}=-2.81\times 10^{-9}$ V Pa${}^{-1}$. Around t∼ 750 s the ramping of *I* is initiated and is seen as a stepwise increase of both *I* and Δϕ with time. It is noted that when Δϕ reaches 0 the EKEC system has reached its maximum streaming current. When Δϕ>0, electrical power is supplied to the flow cell and the system formally operates as a pump. The additional pumping through the membrane has the same direction as the pressure-driven flow. For each 60 s plateau a linear regression is performed and the volume flow through the membrane is calculated as: $q_{V}=dm/dt\rho^{-1}$. For these calculations it is assumed that the permeated solution has the density ρ=1 g cm${}^{-3}$. For *I*, Δϕ, and $\Delta p_{\mathrm{LM}}$ the average value for each plateau was considered for further analysis.

**Figure 2:**
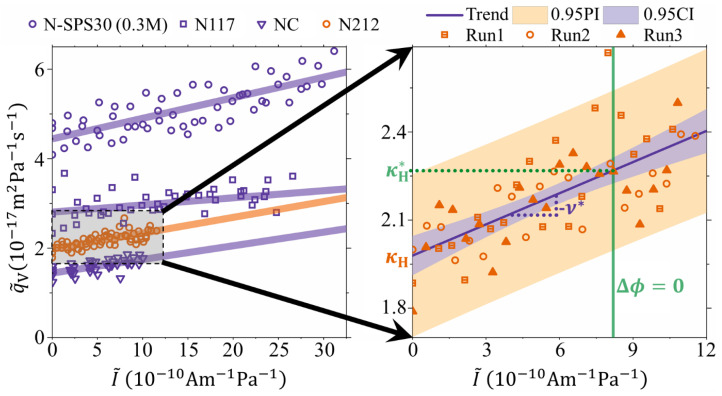
Direct measurement of the electroviscous effect shown as the normalized volumetric membrane flow (${\tilde{q}}_{V}$) versus normalized electrical current ($\tilde{I}$) through the external electrical load. **Left**: Data for 30 wt% SPS in Nafion (N-SPS30: purple circles) in 0.3 M LiI, and for Nafion117 (N117: purple squares), Cast-Nafion (NC: purple triangles), and Nafion212 (N212: orange circles) in 1 M LiI. **Right**: Magnification of the N212 data performed as three repeated experiments under the same conditions. Solid lines are linear fits with ${\tilde{q}}_{V}=\nu^{🟉}I+{\tilde{\kappa }}_{H}$. Light blue area represents the 95% confidence interval. Light red area is the 95% prediction interval for the data points. Green vertical line illustrates the $\tilde{I}$-value where the electrical potential Δϕ=0 across the membrane. The trend-line value at this point equals the intrinsic hydraulic permeability coefficient (${\left[{\tilde{q}}_{V}\right]}_{\Delta \phi =0}=\kappa_{H}^{🟉}$), the *y*-axis intercept equals the hydraulic permeability coefficient (${\left[{\tilde{q}}_{V}\right]}_{I=0}=\kappa_{H}$), and the slope equals the negative streaming potential coefficient ($-\nu^{🟉}$).

Figure 2 shows the normalized volumetric flow (${\tilde{q}}_{V}=q_{V}\Delta xA^{-1}\Delta p^{-1}$) as a function of the normalized current ($\tilde{I}=I\Delta xA^{-1}\Delta p^{-1}$). Here the normalization is with respect to the membrane thickness, active area, and pressure difference ($\Delta xA^{-1}\Delta p^{-1}$). The left-hand side of Figure 2 shows data for several experiments in 1 M LiI/I_2_ solution: with N212 membrane (orange circles), N117 (purple squares), NC (purple triangles) and N-SPS30 membrane (purple circles). The N-SPS30 membrane was tested in a 0.3 M LiI/I_2_ solution in addition to the 1 M test, as to investigate whether the ion selectivity of this membrane with higher a ion channel characteristic dimension was lowered due to the higher electrolyte concentration. It is noted that for each raw dataset a few outliers (≤2) were removed because very large fluctuations of the recorded mass caused unrealistic values of $q_{V}$ over the corresponding 60 s time periods. We attributed these single events to the transients in the hydraulic circuit of small air bubbles that were trapped in the porous electrodes at the beginning of the experiment. The right-hand side of Figure 2 reports a magnification for the N212 membrane. The experiment was repeated three times under identical conditions, and although there is a relatively large scattering, the data is reproducible within the experimental uncertainty. Nonetheless, for all the experiments, a clear linear increase of ${\tilde{q}}_{V}$ with $\tilde{I}$ is seen and is clear experimental evidence of the electroviscous effect not measured directly in ion-exchange membranes before.

It was earlier shown (Equation (8) in Reference [14]) that $\Delta p\Delta x^{-1}=-\kappa_{H}^{-1}q_{\overset{˚}{V}}A-\nu \kappa_{H}^{-1}IA$, which basically is a restatement of one phenomenological transport equation in terms of observable transport coefficients ($\kappa_{H}$ and ν) and has, for this reason, universal applicability for electrokinetic systems. By rearrangement, this equation can be rewritten as: ${\tilde{q}}_{V}=-\nu \tilde{I}+\kappa_{H}$. In this form, the membrane permeated flux is a linear function of the current (electroosmosis) that can either increase or decrease the permeation depending on the direction of the current, with a slope that is the negative streaming potential coefficient. This equation is fitted to the data for all the experiments in Figure 2 and the regression results have been reported as solid lines. The values of $\nu^{🟉}$ and $\kappa_{H}$ obtained from the linear regression are listed in Table 1. Furthermore, from the experimental results it is possible to obtain the values of the streaming current when the cell is short-circuited (Δϕ=0). It is noted that this is not the intrinsic streaming current of the membrane, but that of the membrane/cell assembly. This streaming current is reduced with respect to the intrinsic one because of additional cell and electrode charge transfer resistances [21]. In any case, the vertical green line in Figure 2, shows the magnitude of $\tilde{I}$ at Δϕ=0, which in the case of N212 membranes, is around $8\times 10^{-10}$ A m${}^{-1}$ Pa${}^{-1}$. At this value ${\left[{\tilde{q}}_{V}\right]}_{\Delta \phi =0}=\kappa_{H}^{🟉}$ and it corresponds to the measurement of the hydraulic permeability without electroviscous effects. Again, it is noted that this is for the membrane/cell assembly and the $\kappa_{H}^{🟉}$-value would be larger if cell and charge-transfer resistance can be reduced even further. The values of $\kappa_{H}^{🟉}$ for all four membranes are included in Table 1.

From Table 1 it is seen that $\kappa_{H}$ for all membranes are in the range from about 1.5 to 4.5 $\times 10^{-17}$ m${}^{2}$ Pa${}^{-1}$ s${}^{-1}$ where the lowest value is observed for an NC membrane cast from a Nafion solution while the highest value is seen for the N-SPS30 membrane. This is expected since the N-SPS30 contains an additive (porogen) that increases the characteristic dimension of the ion-channels, thereby increasing the permeability [22]. Additionally, it is seen that the relative electroviscous effect, stated as the ratio $\kappa_{H}^{🟉}\kappa_{H}^{-1}$, does not change significantly among the different membranes. This is also seen from the small variation of the streaming potential coefficient obtained from the slope of the ${\tilde{q}}_{V}$ vs $\tilde{I}$ data, with the exception of the N117 membrane where it appears quite low. These values can also be compared to direct measurement of the streaming potential coefficient (also included in Table 1). In general, the two values agree with each other within the experimental uncertainty, with the exception of the values for the N117 membrane. The EKEC *figure-of-merit* can be found from $\beta =\kappa_{H}^{🟉}\kappa_{H}^{-1}-1$ [14,16,23] and are also reported in Table 1. These can be compared to ones obtained from $\beta =\nu^{2}\sigma^{🟉}\kappa_{H}^{-1}$; the good agreement between the different experimental determinations of β corroborates the figures obtained for the electroviscous effect.

The results for the electroviscous effect presented in this work clearly show that the Nafion and Nafion-based membranes with iec ∼1 meq g${}^{-1}$ despite the differences in thickness (ranging from 60 to 264 μm in dry conditions), preparation method (casting or extrusion), and structure (with or without sacrificial porogen), all have a similar value for $\kappa_{H}^{🟉}\kappa_{H}^{-1}=1.15_{-0.052}^{+0.035}$. Therefore, this latter value can be used as a first approximation for describing the magnitude of the electroviscous effect in Nafion membranes for simple monovalent electrolytes at concentration up to 1 M.

## 4. Conclusions

For the first time in literature, we have obtained direct measurements of the electroviscous effect and the intrinsic hydraulic permeability through ion-selective membranes by varying the imposed ionic current. This was measured for four different Nafion and Nafion-based membranes, two commercial extruded membranes and two cast from commercially available hydroalcoholic solutions. The results have been found to be in good agreement with existing theoretical models previously published with regard to determination of the EKEC efficiency and the transport coefficients of hydraulic permeability and streaming potential. We report here the electroviscous effect for Nafion based membranes to have a value of $\kappa_{H}^{🟉}\kappa_{H}^{-1}=1.15_{-0.052}^{+0.035}$.

## Figures and Tables

**Table 1:** Overview of the obtained transport coefficients for the four different Nafion membranes at various concentrations. Values after ± are standard deviations.

Membrane		N212	N117	NC	N-SPS30	N-SPS30
LiI conc.	M	1	1	1	1	0.3
iec	meq g${}^{-1}$	0.95–1.01${\;}^{a}$	0.95–1.01${\;}^{a}$	0.94 ± 0.03	1.18 ± 0.02	
$\sigma^{🟉\;b}$	S m${}^{-1}$	0.291 ± 0.001	0.637 ± 0.003	0.312 ± 0.006	0.830 ± 0.006	0.462 ± 0.002
Δx	μm	60	175	92	264	264
−ν	$10^{-9}$ V Pa${}^{-1}$	2.81 ± 0.01	2.60 ± 0.10	2.78 ± 0.04	2.76 ± 0.01	3.71 ± 0.01
$-\nu^{🟉}{\;}^{c}$	$10^{-9}$ V Pa${}^{-1}$	3.5 ± 0.4	1.3 ± 0.5	3.0 ± 0.5	3.5 ± 0.7	4.3 ± 0.5
$\kappa_{H}$	$10^{-17}$ m${}^{2}$ Pa${}^{-1}$ s${}^{-1}$	1.98 ± 0.03	2.24 ± 0.07	1.44 ± 0.03	4.32 ± 0.11	4.47 ± 0.09
$\kappa_{H}^{🟉}$	$10^{-17}$ m${}^{2}$ Pa${}^{-1}$ s${}^{-1}$	2.27 ± 0.03	2.46 ± 0.07	1.70 ± 0.03	5.12 ± 0.11	5.12 ± 0.09
$\kappa_{H}^{🟉}\kappa_{H}^{-1}$	-	1.15	1.10	1.18	1.19	1.15
β/$\eta_{\max}{\;}^{d}$	-/%	0.12/2.7	0.19/4.4	0.17/3.9	0.15/3.4	0.14/3.3
β/$\eta_{\max}{\;}^{e}$ from $\nu^{🟉}$	-/%	0.18/4.1	0.05/1.2	0.20/4.5	0.24/5.3	0.19/4.4
β/$\eta_{\max}{\;}^{f}$ from $\kappa_{H}^{🟉}\kappa_{H}^{-1}-1$	-/%	0.15/3.4	0.10/2.3	0.18/4.1	0.19/4.2	0.15/3.4

Notes: ${}^{a}$ from manufacturer specifications. ${}^{b}$ This apparent ion conductivity is calculated as the total ion conductivity of the flow cell: $\sigma^{🟉}=R_{\mathrm{cell}}^{-1}A^{-1}\Delta x$, with $R_{\mathrm{cell}}$ being the ohmic resistance of the flow cell found as the slope of the polarization curve, *A*, and Δx, are the membrane active area and thickness, respectively. ${}^{c}$ This value for the streaming potential coefficient is found as the slope of ${\tilde{q}}_{V}=-\nu \tilde{I}+\kappa_{H}$. ${}^{d,e,f}$ calculations of the figure-of-merit and EKEC maximum efficiency: $\eta_{\max}=\left(\sqrt{\beta +1}-1\right){\left(\sqrt{\beta +1}+1\right)}^{-1}$ by: ${}^{d}$$\beta =\nu^{2}\sigma^{🟉}\kappa_{H}^{-1}$; ^e^$\beta =\nu^{🟉2}\sigma^{🟉}\kappa_{H}^{-1}$; ${}^{f}$$\kappa_{H}^{🟉}\kappa_{H}^{-1}-1$.

## Nomenclature

Symbols	Description	Units
*A*	Membrane active area	m${}^{2}$
*I*	Electrical current	A
$\tilde{I}$	normalized current; $\tilde{I}=I\Delta xA^{-1}\Delta p^{-1}$	$A\;m^{-1}{\mathrm{Pa}}^{-1}$
$p_{H}$, $p_{L}$	Hydrostatic pressure	Pa
$q_{\mathrm{circ}}$	Flow rate	${\mathrm{mL}\;s}^{-1}$
$q_{V}$	Volumetric flow; $q_{V}=dm/dt\rho^{-1}$	${\mathrm{mL}\;s}^{-1}$
${\tilde{q}}_{V}$	normalized volumetric flow; ${\tilde{q}}_{V}=q_{V}\Delta xA^{-1}\Delta p^{-1}$	$m^{2}{\mathrm{Pa}}^{-1}s^{-1}$
Greek symbols		
β	EKEC figure of merit; $\beta =\frac{\nu^{2}\sigma }{\kappa_{H}}$	
Δm	Mass change	kg
Δϕ	Potential across the membrane	V
$\Delta p_{\mathrm{LM}}$	Logarithmic mean pressure difference	Pa
Δx	Membrane thickness	m
$\eta_{\max}$	EKEC maximum efficiency; $\eta_{\max}=\frac{\sqrt{\beta +1}-1}{\sqrt{\beta +1}+1}$	
$\kappa_{H}$	Hydraulic permeability; $\kappa_{H}={\left[{\tilde{q}}_{V}\right]}_{I=0}$	m${}^{2}$ Pa${}^{-1}$ s${}^{-1}$
$\kappa_{H}^{🟉}$	Intrinsic hydraulic permeability; $\kappa_{H}^{🟉}={\left[{\tilde{q}}_{V}\right]}_{\Delta \phi =0}$	m${}^{2}$ Pa${}^{-1}$ s${}^{-1}$
ν	Streaming potential coefficient	$V\;{\mathrm{Pa}}^{-1}$
$\nu^{🟉}$	Intrinsic streaming potential coefficient	$V\;{\mathrm{Pa}}^{-1}$
ρ	Density	kg m${}^{-3}$
σ	Membrane (ionic) conductivity	S m${}^{-1}$
$\sigma^{🟉}$	Apparent membrane (ionic) conductivity	S m${}^{-1}$
